# The relationship between depression, anxiety, and disability in patients with multiple sclerosis: A cross‐sectional study from western Iran

**DOI:** 10.1002/pcn5.70305

**Published:** 2026-02-25

**Authors:** Nazanin Razazian, Mohamad Safaei, Fayeq Bazyar, Dariush Afshari, Sharareh Eskandarieh, Abdorreza Naser Moghadasi, Milad MohamadYari, Mansour Rezaei, Negin Fakhri, Kianoosh Khamooshian

**Affiliations:** ^1^ Neuroscience Research Center, Health Institute Kermanshah University of Medical Sciences Kermanshah Iran; ^2^ Clinical Research Development Center, Imam Reza Hospital Kermanshah University of Medical Sciences Kermanshah Iran; ^3^ Multiple Sclerosis Research Center, Neuroscience Institute Tehran University of Medical Sciences Tehran Iran; ^4^ Social Development and Health Promotion Research Center Kermanshah University of Medical Sciences Kermanshah Iran; ^5^ Clinic of Multiple Sclerosis, Imam Reza Hospital Kermanshah University of Medical Sciences Kermanshah Iran

**Keywords:** anxiety, cross‐sectional studies, depression, disability evaluation, Iran, multiple sclerosis, psychological distress

## Abstract

**Aim:**

Multiple sclerosis (MS) is a chronic demyelinating disease of the central nervous system often accompanied by psychological comorbidities such as depression and anxiety, which may aggravate physical disability. This study aimed to assess the relationship between depression, anxiety, and disability among patients with MS in western Iran.

**Methods:**

A cross‐sectional analytical study was conducted among 180 MS patients attending the Boustan MS Clinic in Kermanshah, Iran. Participants completed the Persian versions of the Beck Depression Inventory (BDI) and Beck Anxiety Inventory (BAI). Disability was evaluated using the Expanded Disability Status Scale (EDSS). Data were analyzed using Spearman correlation and stepwise multiple regression.

**Results:**

The mean age of participants was 41.33 ± 9.09 years, and 75% were female. The mean EDSS score was 3.28 ± 1.56, BDI score 19.87 ± 10.21, and BAI score 13.28 ± 9.96. Depression and anxiety were significantly correlated with disability (*r* = 0.404 and *r* = 0.399, respectively; *p* < 0.01), and with each other (*r* = 0.702, *p* < 0.01). Regression analysis showed that depression alone explained 17.6% of disability variance, which increased to 36.2% when anxiety was added. Lower education level and positive family history of MS were associated with higher psychological distress.

**Conclusion:**

Depression and anxiety are common and strongly associated with greater physical disability in MS patients. Integrating psychological assessment and intervention into MS management may help improve functional outcomes and quality of life.

## INTRODUCTION

Multiple sclerosis (MS) is a chronic inflammatory and demyelinating disorder of the central nervous system that primarily affects young adults and often leads to varying degrees of neurological disability.^1^, ^2^ The disease is characterized by multifocal demyelination, axonal injury, and gliosis, which result in a broad spectrum of neurological symptoms such as motor weakness, fatigue, sensory loss, and visual disturbances.^3^, ^4^ Globally, the prevalence of MS has been increasing, affecting approximately 2.8 million people, with a clear female predominance.^5^, ^6^ In Iran, the incidence has also risen during the past two decades, particularly among women, reflecting both environmental and genetic susceptibilities.^7^, ^8^

Although MS is primarily known for its physical manifestations, its psychological and emotional consequences are equally significant. Depression and anxiety are among the most common psychiatric comorbidities in individuals with MS, with reported prevalence rates ranging from 14% to 54% for depression and around 35% for anxiety.^9^, ^10^, ^11^, ^12^, ^13^ These disorders can appear at any stage of the disease and are often linked to both neurobiological mechanisms and psychosocial stressors.^3^, ^14^, ^15^ The burden of psychiatric symptoms is frequently underestimated in clinical practice, despite their substantial impact on quality of life, social functioning, and treatment adherence.^16^, ^17^

The mechanisms underlying depression and anxiety in MS are multifactorial. Neuroinflammatory cytokines such as interleukin‐6 and tumor necrosis factor‐α, alterations in hypothalamic–pituitary–adrenal axis activity, and serotonergic dysregulation have all been proposed to contribute to these symptoms.^18^ In addition, fatigue, cognitive dysfunction, and uncertainty about disease progression can intensify psychological distress, creating a complex interplay between mental and physical health.^16^

The Expanded Disability Status Scale (EDSS) is the most widely used measure of neurological disability in MS and provides a global index of functional impairment.^19^ Previous research has shown significant correlations between EDSS scores and measures of depression and anxiety, indicating that worsening physical disability is often accompanied by deteriorating emotional health.^9^, ^11^, ^13^, ^16^, ^18^, ^20^, ^21^ Conversely, psychological distress may further exacerbate disability through mechanisms such as reduced motivation, impaired coping, and decreased treatment adherence.^6^, ^10^, ^22^

Despite numerous studies worldwide, the relationship between psychological symptoms and disability has been insufficiently examined in specific cultural contexts such as Iran. In Iran, sociocultural and economic factors—including strong family‐centered caregiving, social stigma toward psychiatric disorders, differences in health literacy, and more limited access to specialized mental health services compared with many Western countries—may substantially influence patients' perception of illness and emotional responses to chronic neurological disease.^7^, ^8^ These contextual factors may affect the recognition, reporting, and management of depression and anxiety in individuals with MS.

Therefore, the present study aims to examine the relationship between depression and anxiety with disability scores among patients with MS attending the Boustan MS Clinic in Kermanshah, Iran. By employing validated Persian versions of the Beck Depression Inventory (BDI) and Beck Anxiety Inventory (BAI)^23^, ^24^ along with the EDSS, this study seeks to clarify how emotional disorders correlate with neurological disability. Identifying these associations will help clinicians integrate psychological screening into MS management, improve quality of life, and support more effective disease control.

## MATERIALS AND METHODS

### Study design

This research was designed as a cross‐sectional analytical study to investigate the relationship between depression, anxiety, and disability in patients with MS. The study was conducted as a single‐center investigation at the comprehensive MS Clinic of Boustan in Kermanshah, Iran, after obtaining ethical approval from the Research Ethics Committee of Kermanshah University of Medical Sciences.

### Participants and sampling

The study population included all patients diagnosed with various types of MS who attended the MS clinic during the study period. The diagnosis of MS was confirmed by a neurologist based on the McDonald criteria.^25^

The inclusion criteria were: Definite diagnosis of MS, age between 18 and 60 years, ability to provide informed consent, and absence of other major psychiatric or neurological disorders.

The exclusion criteria consisted of the following: unwillingness to participate, cognitive impairment preventing questionnaire completion, relapse at the time of study assessment, and the presence of other major psychiatric or neurological disorders (such as psychotic disorders or severe neurocognitive disorders). Patients with depression or anxiety were not excluded, as these conditions were the main focus of the study.

The minimum sample size was determined based on the prevalence of anxiety among MS patients (35.7%) reported by Korostil and Feinstein,^26^ using a confidence level of 95% and a margin of error of 7%. Accordingly, the minimum number of participants required was estimated to be 180 patients.

Patients with MS were initially identified through the National Multiple Sclerosis Registry of Iran (NMSRI)^27^ as registered patients affiliated with the Boustan MS Clinic in Kermanshah. Eligible patients who attended the clinic during the study period were consecutively approached and invited to participate. A convenience sampling method was used, and recruitment continued until the predetermined sample size of 180 participants was achieved.

### Data collection tools

#### Demographic and clinical questionnaire

A researcher‐designed questionnaire was used to collect demographic and clinical data, including age, sex, education level, marital status, employment status, disease duration, age at diagnosis, family history of MS, and current disease‐modifying therapy.

#### Expanded Disability Status Scale

The EDSS is a disease‐specific scale developed to assess neurological disability in patients with MS.^19^ The scale ranges from 0 to 10, with higher scores indicating greater MS‐related disability. The EDSS was evaluated by a neurology resident trained in standardized scoring methods. For descriptive purposes, EDSS scores were categorized as mild (0–3.5), moderate (4.0–5.5), and severe (≥6.0) disability, consistent with commonly used clinical classifications in MS research.^19^

#### Beck Depression Inventory

The BDI, developed by Beck and colleagues,^28^ consists of 21 items, each rated on a 4‐point Likert scale from 0 to 3, yielding a total score range of 0–63. Scores of 0–13 indicate minimal or no depression, 14–19 mild depression, 20–28 moderate depression, and ≥29 severe depression. The Persian version of the BDI has been validated in Iranian populations and shows high internal consistency (Cronbach's *α* = 0.87) and test–retest reliability (*r* = 0.74).^24^

#### Beck Anxiety Inventory

The BAI is another 21‐item self‐report scale used to measure anxiety severity.^29^ Each item is scored from 0 (“not at all”) to 3 (“severely—it bothered me a lot”), with total scores ranging from 0 to 63. Scores of 0–7 indicate minimal anxiety, 8–15 mild anxiety, 16–25 moderate anxiety, and ≥26 severe anxiety. The Persian version of the BAI has been validated in Iranian populations and demonstrates strong internal reliability (Cronbach's *α* = 0.92) and test–retest reliability (*r* = 0.72).^23^

### Data collection procedure

After obtaining consent, participants were interviewed by a trained psychologist who administered the BDI and BAI questionnaires in a private setting. Demographic and clinical information was recorded using the structured questionnaire. Disability was independently assessed by a trained neurology resident using the EDSS. Each patient was assigned a unique identification code to ensure confidentiality. All assessments were completed during a single clinical visit.

### Statistical analysis

All statistical analyses were planned a priori in accordance with the predefined study objectives. All data were analyzed using spss software version 27.0 (IBM Corp., Armonk, NY, USA). Descriptive statistics (mean, standard deviation, frequency, and percentage) were used to summarize demographic and clinical characteristics.

Before performing inferential analyses, the Kolmogorov–Smirnov test was applied to verify the normality of quantitative data distribution. Disability measured by the EDSS was defined as the primary outcome variable, while depression (BDI) and anxiety (BAI) were considered independent variables. For inferential analysis, the Spearman correlation coefficient was used to determine the relationship between depression, anxiety, and EDSS scores; the Mann–Whitney *U* and Kruskal–Wallis tests were used for group comparisons by demographic variables; and a stepwise multiple regression model was employed to predict disability score based on depression and anxiety levels.

A *p*‐value of less than 0.05 was considered statistically significant.

### Ethical considerations

Written informed consent was obtained from all participants after providing clear information about the study's purpose and procedures. All data were kept confidential and anonymized. Participants with clinically significant depression or anxiety were referred for free psychological consultation with the study's clinical psychologist. The research protocol was approved by the Ethics Committee of Kermanshah University of Medical Sciences (ethics code: IR.KUMS.MED.REC.1402.154).

## RESULTS

### Demographic and clinical characteristics

A total of 180 patients with MS participated in this study. The mean age was 41.33 ± 9.09 years, and the majority were female (75%), corresponding to a female‐to‐male ratio of 3:1. The average disease duration was 9.73 ± 6.97 years. Most participants were married (70%) and had a diploma or university education (73.9%). The relapsing–remitting type (RRMS) was the predominant clinical form (72.2%), followed by primary progressive (16.7%) and secondary progressive (10.0%) types. Approximately 16.7% of patients reported a positive family history of MS.

Detailed demographic and clinical data are presented in Table 1.

**Table 1:** Demographic and clinical characteristics of patients with multiple sclerosis (*n* = 180).

Variable	Category/mean ± SD	Frequency (*n*)	Percentage (%)
Age (years)	41.33 ± 9.09	—	—
Sex	Male	45	25.0%
	Female	135	75.0%
Marital status	Single	53	29.4%
	Married	126	70.0%
Education level	Elementary/high school	47	26.1%
	Diploma/university	133	73.9%
Family history of MS	Positive	30	16.7%
	Negative	150	83.3%
Disease duration (years)	9.73 ± 6.97	—	—
Type of MS	RRMS	130	72.2%
	PRMS	1	0.6%
	SPMS	18	10.0%
	PPMS	30	16.7%

Abbreviations: MS, multiple sclerosis; PPMS, primary progressive MS; PRMS, progressive relapsing MS; RRMS, relapsing‐remitting MS; SD, standard deviation; SPMS, secondary progressive MS.

### Disability, depression, and anxiety levels

The mean EDSS score among participants was 3.28 ± 1.56, indicating that most patients were in the mild to moderate stages of functional limitation. As shown in Table 2, 74.4% had mild disability, 21.7% had moderate, and 3.9% had severe disability.

**Table 2:** Distribution of disability, depression and anxiety scores.

Variable	Classification/range	Frequency	Percentage	Mean ± SD
EDSS (disability)	Mild (0–3.5)	134	74.4%	3.28 ± 1.56
	Moderate (4.0–5.5)	39	21.7%	
	Severe (≥6)	7	3.9%	
BDI (depression)	Normal (0–13)	87	48.3%	19.87 ± 10.21
	Mild (14–19)	19	10.6%	
	Moderate (20–28)	32	17.8%	
	Severe (≥29)	42	23.3%	
BAI (anxiety)	Normal (0–7)	59	32.8%	13.28 ± 9.96
	Mild (8–15)	63	35.0%	
	Moderate (16–26)	38	21.1%	
	Severe (≥26)	20	11.1%	

Abbreviations: BAI, Beck Anxiety Inventory; BDI, Beck Depression Inventory; EDSS, Expanded Disability Status Scale; SD, standard deviation.

Based on the BDI, the mean depression score was 19.87 ± 10.21, with 23.3% of patients exhibiting severe depression and 17.8% showing moderate depression. Similarly, the mean BAI score was 13.28 ± 9.96, with moderate to severe anxiety observed in 32.2% of participants. These results highlight the high prevalence of psychological symptoms among MS patients in this sample.

Patients with lower educational attainment demonstrated higher mean scores for both depression and anxiety (*p* < 0.001 and *p* = 0.024, respectively). Additionally, those with a positive family history of MS showed higher mean anxiety levels (*p* = 0.004). However, no significant differences were observed across gender or marital status groups.

### Correlations between psychological variables and disability

**Figure 1:**
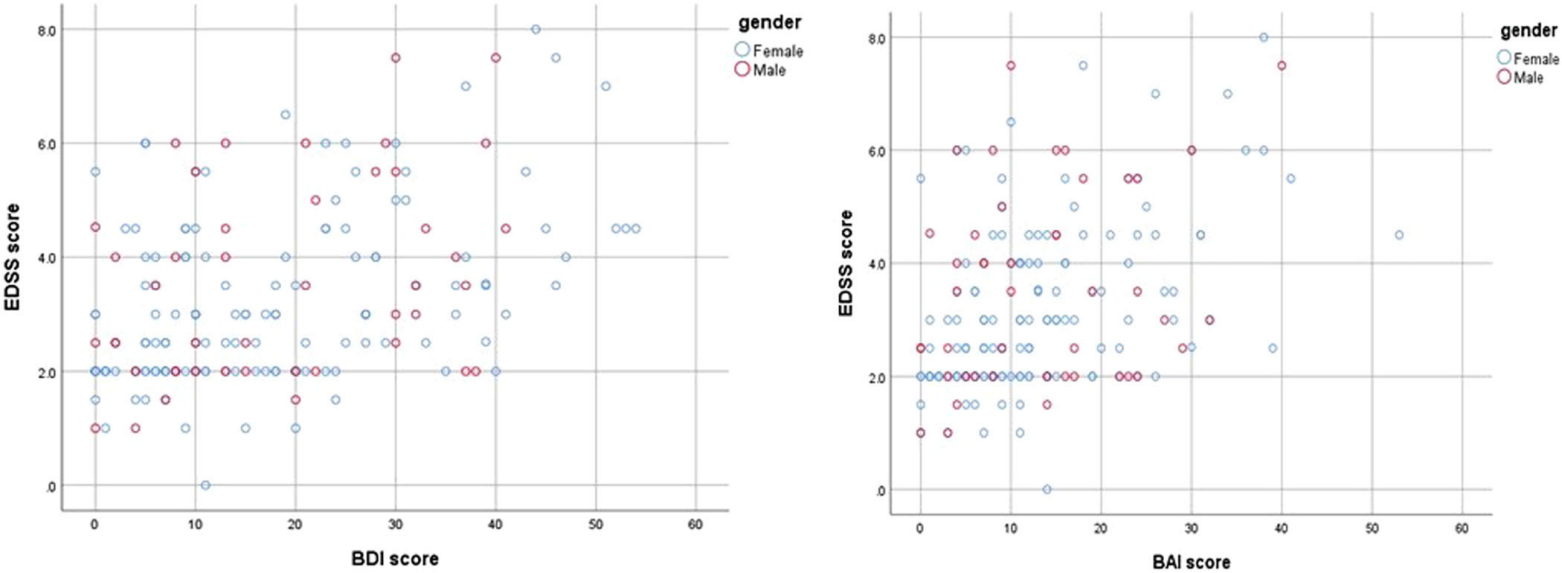
Correlation between psychological distress and disability in patients with multiple sclerosis. (A) Relationship between depression (Beck Depression Inventory [BDI]) and disability (Expanded Disability Status Scale [EDSS]). (B) Relationship between anxiety (Beck Anxiety Inventory [BAI]) and disability (EDSS).

Disability measured by EDSS was treated as the primary outcome variable, and its associations with depression (BDI) and anxiety (BAI) were examined. Correlation analysis using Spearman's rho revealed significant positive relationships between psychological and functional measures. As shown in Table 3, there was a moderate positive correlation between depression (BDI) and disability (EDSS) (*r* = 0.404, *p* < 0.01), and between anxiety (BAI) and disability (EDSS) (*r* = 0.399, *p* < 0.01). Depression and anxiety scores were also significantly correlated with one another (*r* = 0.702, *p* < 0.01). These relationships are illustrated in Figure 1, which presents scatterplots depicting the linear association between BDI, BAI, and EDSS scores. The regression trendlines confirm that higher depression and anxiety levels are associated with increased disability severity.

**Table 3:** Correlations between depression, anxiety, and disability (Spearman's rho).

Variables	*r* (correlation coefficient)	*p*‐value	Interpretation
Depression (BDI)–disability (EDSS)	0.404	<0.01	Significant positive correlation
Anxiety (BAI)–disability (EDSS)	0.399	<0.01	Significant positive correlation
Depression (BDI)–anxiety (BAI)	0.702	<0.01	Significant positive correlation

*Note*: All correlations are statistically significant at the 0.01 level (two‐tailed).

Abbreviations: BAI, Beck Anxiety Inventory; BDI, Beck Depression Inventory; EDSS, Expanded Disability Status Scale.

### Regression analysis

A stepwise multiple regression model was applied to identify predictors of disability (EDSS). As summarized in Table 4, depression alone explained 17.6% (*R* ^2^ = 0.176) of the variance in disability scores (*p* < 0.001). When anxiety was added to the model, the explained variance increased to 36.2% (*R* ^2^ = 0.362), indicating that both depression and anxiety were significant predictors of physical disability in MS patients (*p* < 0.001). Although depression and anxiety were strongly correlated (*r* = 0.702), both variables independently contributed to the regression model, suggesting that each was associated with disability despite their conceptual overlap.

**Table 4:** Stepwise multiple regression predicting disability (Expanded Disability Status Scale [EDSS]) from depression and anxiety.

Model	Predictor variables	*R* ^2^	Adjusted *R* ^2^	*p*‐value	Interpretation
Model 1	Depression only	0.176	0.172	<0.001	Depression explains 17.6% of disability variance
Model 2	Depression + anxiety	0.362	0.347	<0.001	Adding anxiety raises explained variance to 36.2%

*Note*: Regression analysis shows both depression and anxiety significantly predict disability, with depression being the stronger individual predictor.

Depression emerged as the stronger individual predictor, suggesting that mood disturbances may have a more substantial impact on perceived or actual functional impairment compared to anxiety.

## DISCUSSION

The present study examined the relationship between depression, anxiety, and disability among patients with MS using standardized psychometric and clinical assessment tools. The results demonstrated significant positive correlations between both depression and anxiety with disability scores, indicating that patients with higher psychological distress tend to experience greater functional impairment. These findings are consistent with several prior studies that have reported similar associations between psychological comorbidities and physical disability in MS populations.^18^, ^20^, ^21^, ^22^, ^30^, ^31^, ^32^

In this study, depression emerged as a stronger predictor of disability compared to anxiety. This observation aligns with findings by Gill et al.,^20^ who reported that depressive symptoms have a more substantial negative impact on functional outcomes in MS patients than anxiety symptoms. Similarly, Curatoli et al.^32^ identified disability level as an independent predictor of both depression and anxiety severity, suggesting that worsening neurological impairment may exacerbate psychological distress through mechanisms such as reduced autonomy, fatigue, and social withdrawal.

Several mechanisms have been proposed to explain the bidirectional relationship between psychiatric symptoms and MS disability. Neuroinflammatory processes involving cytokines such as TNF‐α, IL‐6, and GM‐CSF have been implicated in both MS pathophysiology and mood regulation.^2^, ^4^, ^17^, ^31^ These inflammatory mediators may contribute to neurodegeneration and altered neurotransmitter signaling, leading to increased susceptibility to depression and anxiety.^17^, ^18^ Conversely, the presence of persistent depressive symptoms can impair treatment adherence, reduce engagement in physical activity, and increase fatigue, all of which can accelerate disability progression.^20^, ^22^, ^32^

Consistent with the findings of Korostil and Feinstein,^26^ the prevalence of anxiety among participants in this study was high, with approximately 35% exhibiting mild, 21% moderate, and 11% severe anxiety. Similarly, depressive symptoms were identified in more than 51% of patients, with 23% classified as severely depressed. These rates exceed those observed in the general population and are comparable to previous Iranian studies, such as Karimi et al.,^33^ who reported high levels of psychological distress among MS patients in Kermanshah province. Such findings highlight the need for routine mental health screening within neurological care settings.

Sociodemographic factors also influenced psychological outcomes. Patients with lower educational attainment exhibited significantly higher depression and anxiety scores, supporting the view that education level serves as a buffer against emotional distress by enhancing coping resources and social support.^34^, ^35^ Likewise, individuals with a positive family history of MS had higher mean anxiety scores, possibly due to heightened fear of disease progression or familial illness awareness. However, no significant associations were observed between gender and depression or anxiety levels, which contrasts with some studies reporting higher psychological burden in women.^12^, ^13^ An important contribution of the present study is its focus on patients with MS within the Iranian sociocultural and healthcare context. In Iran, family‐centered caregiving plays a prominent role in the management of chronic diseases, which may both buffer psychological distress through social support and, in some cases, increase emotional burden due to dependency and role changes. Additionally, stigma surrounding mental health disorders remains relatively prevalent, potentially leading to underrecognition and undertreatment of depression and anxiety among MS patients. Limited access to specialized psychological services in certain regions, along with variability in mental health literacy, may further exacerbate psychological distress and its impact on functional outcomes. These contextual factors may partly explain the observed associations between psychological symptoms and disability in this population and highlight the importance of culturally sensitive, multidisciplinary approaches to MS care in Iran.

From a clinical standpoint, the observed positive correlations between EDSS and both depression and anxiety (*r* = 0.404 and *r* = 0.399, respectively) underscore the close interaction between physical and psychological health domains in MS. The stepwise regression model, which explained 36.2% of the variance in disability, further supports the predictive role of psychological factors in functional outcomes. These results are in agreement with Curatoli et al.,^32^ who demonstrated that depression affects fatigue, pain, and treatment adherence, thereby influencing disease progression and quality of life.

The strong correlation observed between depression and anxiety scores reflects the well‐documented overlap between these psychological constructs in individuals with chronic neurological diseases. This raises the possibility of multicollinearity in regression analyses. However, despite this correlation, both depression and anxiety independently contributed to the prediction of disability in the present model, suggesting that each captures a distinct aspect of psychological distress relevant to functional impairment. Nevertheless, these findings should be interpreted with caution, and future studies using longitudinal designs or advanced modeling approaches may help further disentangle the independent effects of depression and anxiety on disability progression.

The present study's findings reinforce the importance of early psychological assessment and intervention in MS management. Implementing psychosocial support programs, cognitive–behavioral therapy, and stress reduction interventions may help alleviate depressive and anxiety symptoms, improve quality of life, and potentially mitigate disability progression.^6^ Given the bidirectional nature of the relationship between emotional distress and physical impairment, addressing mental health in MS care is not merely supportive but an essential component of disease management. From a clinical perspective, the findings of this study suggest that routine psychological screening should be incorporated into standard MS care. Early identification of depression and anxiety using brief validated instruments may allow timely intervention. Evidence‐based psychological treatments such as cognitive–behavioral therapy, psychoeducation, and stress management interventions may help reduce psychological distress and potentially mitigate disability progression. Furthermore, multidisciplinary care models integrating neurological and psychiatric services could improve treatment adherence, functional outcomes, and overall quality of life in patients with MS.

### Study limitations

Several limitations should be considered when interpreting the results of this study. First, the cross‐sectional design precludes any causal inference regarding the relationship between psychological distress and disability in patients with MS. Second, this study was conducted at a single clinical center, which may limit the generalizability of the findings to other populations or healthcare settings. Third, disability was assessed using the total EDSS score, and functional system subscales were not analyzed separately, potentially limiting the evaluation of domain‐specific disability. In addition, cognitive function was not formally assessed using standardized neuropsychological instruments and therefore could not be included in the analysis, despite its known association with psychological distress in MS. Finally, although information on disease‐modifying therapies was collected, medication type and treatment duration were not incorporated into the regression models. Future multicenter and longitudinal studies incorporating detailed cognitive assessments and treatment‐related factors are warranted to better clarify the complex relationships between psychological distress and disability progression in MS.

## CONCLUSION

This study demonstrates a clear association between psychological distress, including depression and anxiety, and disability among patients with MS. Higher levels of depression and anxiety were associated with greater functional impairment, with depression emerging as the stronger predictor. Educational level and family history also influenced psychological outcomes, highlighting the interplay between social and biological factors.

These findings emphasize the need for integrated multidisciplinary approaches to MS management that incorporate psychological assessment and support alongside neurological care. Early identification and treatment of depression and anxiety can play a critical role in improving overall patient outcomes, enhancing treatment adherence, and maintaining quality of life.

In summary, psychological factors are not only consequences of MS‐related disability but also important contributors to disease progression. Incorporating mental health services into MS care frameworks is therefore essential to ensure holistic and effective management for individuals living with this chronic neurological condition. Implementing targeted psychological interventions alongside neurological care may represent an essential strategy for improving functional outcomes in individuals with MS.

## AUTHOR CONTRIBUTIONS

Nazanin Razazian, Dariush Afshari, and Abdorreza Naser Moghadasi designed the study. Mohamad Safaei, Fayeq Bazyar, and Kianoosh Khamooshian collected the data. Milad MohamadYari and Mansour Rezaei performed the statistical analysis. Negin Fakhri and Sharareh Eskandarieh contributed to data interpretation. Milad MohamadYari drafted the manuscript. All authors critically reviewed the manuscript and approved the final version.

## CONFLICT OF INTEREST STATEMENT

The authors declare no conflicts of interest.

## ETHICS APPROVAL STATEMENT

The study protocol was approved by the Ethics Committee of Kermanshah University of Medical Sciences (ethics code: IR.KUMS.MED.REC.1402.154).

## PATIENT CONSENT STATEMENT

Written informed consent was obtained from all participants before enrollment.

## CLINICAL TRIAL REGISTRATION

N/A.

## Data Availability

The data that support the findings of this study are available on request from the corresponding author. The data are not publicly available due to privacy or ethical restrictions.
